# Comparative effectiveness of low-dose CT lung cancer screening among high-risk non-smoking female subgroups and males in China

**DOI:** 10.3389/fonc.2025.1755209

**Published:** 2026-01-15

**Authors:** Yumeng Ding, Le Wang, Wanting Ren, Weiwei Gong, Lingbin Du, Xiangdong Cheng

**Affiliations:** 1 School of Public Health, Nanjing Medical University, Nanjing, China; 2 Department of Cancer Prevention, Zhejiang Cancer Hospital, Hangzhou Institute of Medicine (HIM), Chinese Academy of Sciences, Hangzhou, China; 3 Zhejiang Provincial Centre for Disease Control and Prevention, Hangzhou, China; 4 Department of Gastric Surgery, Zhejiang Cancer Hospital, Hangzhou Institute of Medicine (HIM), Chinese Academy of Sciences, Hangzhou, China

**Keywords:** LDCT screening, lung cancer, non-smoking females, risk stratification, sex-disparity

## Abstract

**Objective:**

Lung cancer screening guidelines abroad predominantly rely on smoking pack-years and age, Chinese guidelines uniquely incorporate non-smoking risk factors, though empirical validation remains limited. This study compared low-dose computed tomography (LDCT) screening participation and effectiveness between non-smoking females and males in real-world settings.

**Methods:**

This study used data from the Cancer Screening Program in Urban China (CanSPUC) in Zhejiang Province, spanning from April 2019 to December 2024. Eligible participants were aged 45 to 74 and were evaluated for a high risk for lung cancer. We stratified the population into three comparison groups: non-smoking females, non-smoking males and smoking males. Four key screening metrics were compared between groups: LDCT adherence rate, positive rate, lung cancer detection rate and false-positive rate. Multivariable robust (modified) Poisson regression was used to estimate adjusted risk ratios (aRRs) between groups.

**Results:**

A total of 88,797 participants aged 45 to 74 years were included in this study, comprising 43,604 non-smoking females and 45,193 males (6988 non-smokers). The overall LDCT participation rate was 61.2%, with a positive rate of 5.6%, lung cancer detection rate was 1.5%, and a false-positive rate of 95.2%. After adjustment for age, BMI, educational level, passive smoking, occupational exposure to hazardous substances, history of tuberculosis; history of COPD; family history of lung cancer, hypertension; hyperlipidemia and diabetes, smoking males showed significantly higher rates than non-smoking females in LDCT adherence (aRR=1.22, 95% CI 1.20-1.24), positive findings (aRR=1.12, 95% CI 1.04-1.20), and lung cancer detection (aRR=1.36, 95% CI 1.17-1.57). Conversely, non-smoking males demonstrated lower adherence (aRR=0.82, 95% CI 0.79-0.85), fewer positive findings (aRR=0.83, 95% CI 0.70-0.98), and reduced lung cancer detection (aRR=0.57, 95% CI 0.37-0.86) compared to non-smoking females. False-positive rates showed no significant differences across groups. During risk stratification of non-smoking females, both passive smoking (aRR=1.46, 95% CI 1.08-2.01) and hormonal abnormalities (aRR=1.39, 95% CI 1.11-1.75) were identified as independent risk factors for lung cancer, with no significant interaction observed between these factors. Multivariable analyses revealed that high-risk non-smoking females with both passive smoking and hormonal abnormalities showed no statistically significant differences in lung cancer detection rates (vs. all males: aRR=0.93, 95% CI 0.77–1.13; vs. smoking males: aRR=0.88, 95% CI 0.72–1.07) or false-positive rates (vs. all males: aRR=0.95, 95% CI 0.85–1.07; vs. smoking males: aRR=0.96, 95% CI 0.85–1.07) compared to male populations.

**Conclusions:**

In summary, current lung cancer screening criteria demonstrate limited applicability in female populations, as evidenced by significantly lower LDCT adherence, positive rates, and detection rates among non-smoking females compared to smoking males. Critically, we identified a well-defined subgroup of non-smoking females characterized by combined passive smoking and hormonal abnormalities. This subgroup exhibited screening outcomes that were not statistically significantly different from those observed in male populations. These findings support the development of more precise, risk-stratified screening criteria that better account for sex-specific differences in lung cancer risk and clinical presentation.

## 1 Introduction

Lung cancer is the leading cause of cancer-related incidence and mortality worldwide. In 2022, approximately 2.5 million new cases and 1.8 million deaths were attributed to lung cancer globally (1). The incidence rate of lung cancer in males is approximately double that in females, but the gender gap is narrowing, with an annual decreasing rate of 7.4% for the age-standardized incidence rate (ASIR) of lung cancer in males and an increasing of 0.9% in females (2). Notably, the rising incidence of lung cancer among females is particularly evident in Asia, where Chinese females experience an alarming annual increase of 8.3% (3).

Evidence from observational studies indicates that passive smoking, radon, PM2.5, family history of lung cancer, and hormonal abnormalities elevates lung cancer risk among non-smokers (4, 5), however, causal inference warrants further validation through higher-quality studies. Low-dose computed tomography (LDCT) screening can reduce lung cancer mortality by 20% to 24% for heavy smoking population (6, 7). However, the epidemiological landscape of lung cancer in China presents distinct characteristics, with a notably rising incidence among non-smoking female (4). China’s lung cancer screening guidelines have accordingly expanded beyond smoking to incorporate additional risk factors including occupational exposures, passive smoking, chronic obstructive pulmonary disease (COPD), and family history of lung cancer (8–10). Nevertheless, the evidence supporting these non-smoking risk factors remains limited and real-world screening effectiveness requires further validation. Critical questions persist regarding the feasibility of implementing LDCT screening in non-smoking female populations, including screening adherence, positive rates, lung cancer detection rates, and potential overdiagnosis risks.

Emerging Asian studies revealed critical insights into the effectiveness of LDCT screening in non-smokers. A recent-published JAMA study reported comparable lung cancer detection rates between Chinese non-smokers (1.6%) and smokers (2.0-2.3%), with non-smokers showing superior early-stage detection (93.2% vs. 80.4%) (11). The Taiwan Lung Cancer Screening for Non-Smokers Trial (TALENT) in China implemented enhanced risk stratification incorporating nonsmoking-specific factors, demonstrating a 2.6% baseline lung cancer detection rate within first-year follow-up, exceeding the 1.1% in the NLST (12). Higher detection rates of invasive lung cancer among non-smoking females were also reported by the Female Asian Never-Smoker Screening Study (FANSS) and Korean cohort study (13, 14). These findings collectively substantiate the clinical feasibility of LDCT implementation in non-smokers. However, the higher detection rate of lung cancer may raise concerns about overdiagnosis. Xu et al.’s investigation revealed substantial overdiagnosis of lung adenocarcinoma in Chinese populations attributable to LDCT screening, particularly among non-smoking females. Overdiagnosis rates exhibited temporal escalation from 22% in 2011–2015 to 50% in 2016-2020 (15). This potential for overdiagnosis exposes patients to the risks of superfluous interventions and consequent psychological distress. Thus, the feasibility of LDCT screening in non-smokers necessitates framing its implementation within the ongoing international discourse on its risk-benefit profile. A critical priority arising from this discourse is the development of more precise risk stratification strategies.

The Cancer Screening Program in Urban China (CanSPUC) was initiated in 2012 as a government-funded, high-risk-based urban screening program spanning 28 provinces. It targets five major cancers: lung, breast, colorectal, liver, and upper gastrointestinal (esophageal and gastric). This initiative was designed to implement an integrated approach combining primary, secondary, and tertiary prevention strategies to holistically enhance population health. Based on the results of a cancer risk assessment questionnaire, participants are categorized according to their risk level. High-risk individuals are then advised to undergo corresponding cancer screenings, with those at high risk for lung cancer receiving LDCT scans at appointed tertiary hospitals.

In this study, we reported the outcomes of lung cancer screening in Zhejiang Province from the Cancer Screening Program in Urban China (CanSPUC) between April 2019 and December 2024. We compared the performance of non-smoking females and males across key screening metrics, including LDCT adherence, positive rate, lung cancer detection rate, and false positive rate. This study addresses two critical objectives: first, to evaluate the current screening guidelines by assessing whether non-smoking females derive comparable screening benefits to males; and second, to refine risk stratification by identifying higher-risk subgroups among non-smoking females and comparing screening efficiency and yield across risk profiles. The aim of this study is to provide evidence for the formulation of tailored and effective lung cancer screening strategies in the future.

## 2 Materials and methods

### 2.1 Study design and data source

**Figure 1:**
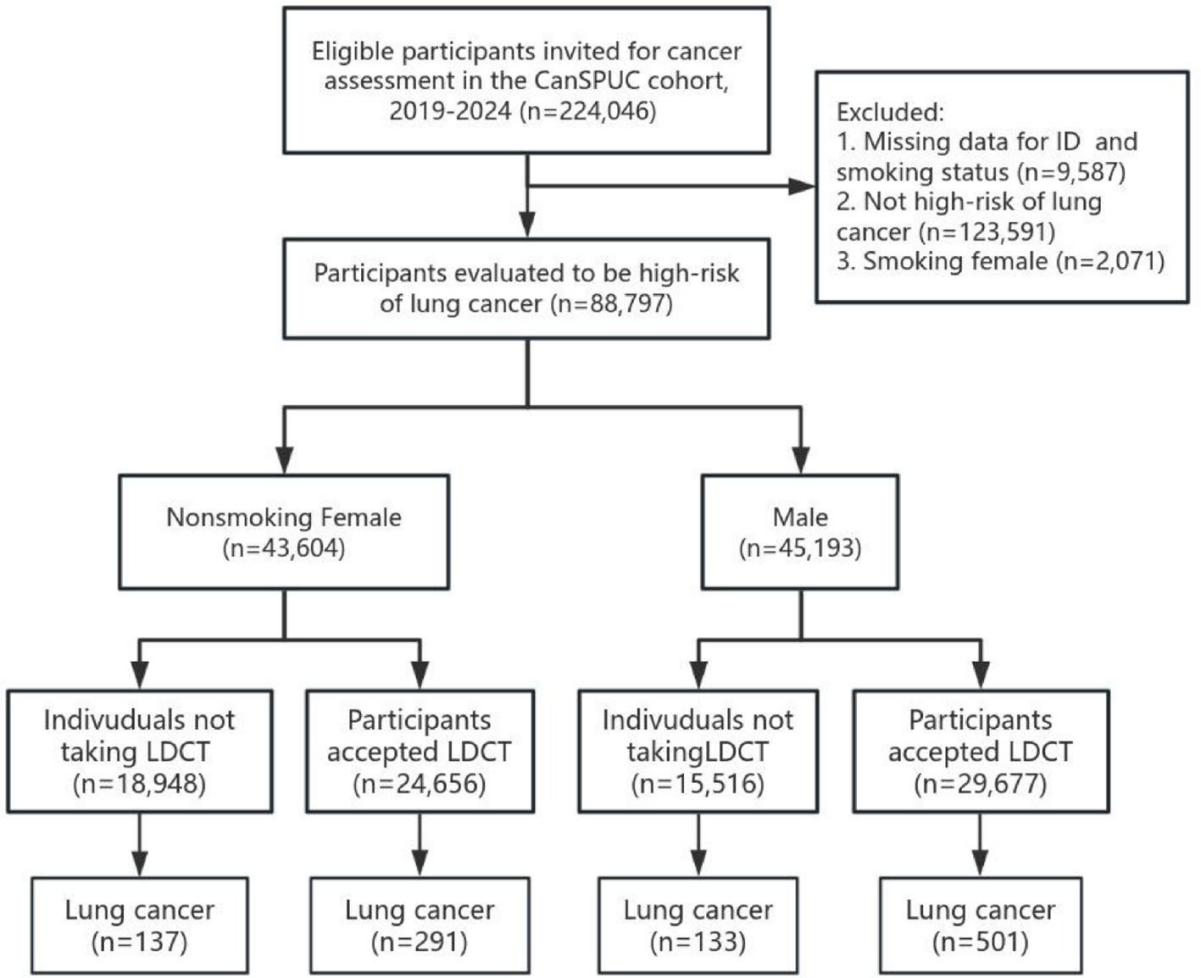
Flow diagram of participants recruitment in CanSPUC cohort, 2019-2024. CanSPUC, Cancer Screening Program in Urban China.

We performed a cross-sectional study under the framework of the CanSPUC program. Residents in Zhejiang provinces aged 45-74, asymptomatic of lung cancer, without taking other cancer screening programs in the past 5 years were eligible for the CanSPUC program. Data collection was performed using a standardized Cancer Risk Assessment Questionnaire, which was administered by trained interviewers via telephone or face-to-face interviews. All collected data were entered into a centralized data management system. To ensure data quality, automated consistency checks were implemented, and each participant was assigned a unique identifier to facilitate the linkage and tracking of all subsequent information. After entering the CanSPUC cohort, individuals who meet one of the following criteria will be recommended to undertake LDCT screening: (a) ever-smokers with at least a 20 pack-year smoking history who are either current smokers or former smokers who have quit smoking within the past 15 years; (b) a diagnosis of chronic obstructive pulmonary disease (COPD) or a history of diffuse interstitial pulmonary fibrosis; (c) a history of exposure to one or more of eleven occupational carcinogens (asbestos, radon, beryllium, uranium, chromium, cadmium, nickel, silicon, diesel exhaust, soot, or soot ash) for more than five years; (d) non-smoking females exposed to second-hand smoke from a family member living in the same household or a co-worker working in the same room who meets the criteria in (a) above. Both the high-risk non-smoking female and the high-risk male were involved in the current study. A flow diagram showing the recruitment of the study population is shown in **Figure 1**.

Data on lung cancer related examinations and clinical outcomes were sourced from hospital medical records, the regional health insurance system of Zhejiang Province, and the Zhejiang Provincial Cancer Registry. All data were transmitted securely to the coordinating center at the National Cancer Center (NCC) via the web-based management system of the National Cancer Prevention and Control Network (NCPCN). The program obtained approval from the ethics committees of the National Cancer Center of China/Cancer Hospital, Chinese Academy of Medical Sciences and Peking Union Medical College, as well as the ethics committees from the involved hospitals.

### 2.2 Outcomes definition

The study outcomes were 1) LDCT adherence, defined as the proportion of all individuals identified as high-risk for lung cancer who completed an LDCT screening within 3 months after the risk assessment date; 2) LDCT positive rate, defined as the proportion of high-risk individuals who underwent LDCT screening with a positive result, where a positive finding included suspected lung cancer or high-risk positive nodules (solid or part-solid nodules ≥8 mm; non-solid nodules ≥15 mm); 3) Lung cancer detection rate, defined as the number of lung cancer cases per 1,000 high-risk individuals screened by LDCT, as confirmed by pathological biopsy (the gold standard); 4) False positive rate, defined as the proportion of individuals with positive LDCT results who were confirmed not to have lung cancer through gold-standard verification or subsequent follow-up. Participants with positive screening results who lacked conclusive verification were excluded from this analysis, which may introduce selection bias if these cases are not missing at random.

### 2.3 Variables definition

Factors studied included age; body mass index (defined as weight in kilograms divided by the square of height in meters); educational level; passive smoking (as detailed in criterion 4 of the Lung Cancer Risk Assessment section under “Study Design and Data Source”); occupational exposure to hazardous substances; family history of lung cancer; history of COPD; hypertension; hyperlipidemia and diabetes.

Regarding female reproductive factors, this study included five variables: (1) age at menarche, with <13 years and >16 years defined as abnormal; (2) age at menopause, with <43 years and >52 years defined as abnormal; (3) reproductive span, defined as age at menopause minus age at menarche, with <30 years and >35 years considered abnormal; (4) age at first live birth, with <20 years and >25 years defined as abnormal; and (5) duration of breastfeeding, with <7 weeks and >12 weeks defined as abnormal. Females with ≥2 abnormal indicators were defined as having hormonal abnormalities, a cutoff that was validated as optimal through sensitivity analysis. The cutoff values for abnormal female reproductive factors were referenced from the CKB database (16).

### 2.4 Statistical analysis

In descriptive analyses of the study population’s characteristics, we used percentages, means, and standard deviations (SDs) to describe the overall cohort and by study group (non-smoking female group and male group). Given the binary nature of our primary outcomes and for direct estimation of risk ratios (RRs), we employed multivariable robust (modified) Poisson regression models to generate adjusted RRs (aRRs) (17). This approach was selected because the relative risk (RR) is a more direct and clinically interpretable measure of effect than the odds ratio (OR) for our study outcomes. Specifically, when the outcome is not rare (e.g., our outcomes of screening adherence and positive rate exceed 10%), the OR derived from logistic regression can substantially overestimate the RR. Modified Poisson regression avoids this overestimation by providing a direct and robust estimate of the RR. Risk ratios were adjusted for age, BMI, educational level, passive smoking, occupational exposure to hazardous substances, history of tuberculosis; history of COPD; family history of lung cancer, hypertension; hyperlipidemia and diabetes. While this adjustment strengthens the internal validity of our estimates, we acknowledge the potential for residual confounding due to unmeasured or imperfectly measured factors (e.g., genetic predisposition, indoor air pollution). To contextualize our findings, we discuss the potential impact of such confounding on the interpretation of the results. All statistical analyses and graphical visualizations were performed using R 4.4.1. All tests were 2-sided, and *P* ≤ 0.05 was considered to be statistically significant.

## 3 Results

### 3.1 Characteristics of the study population

As is shown in **Figure 1**, there were 224,046 eligible participants recruited in the CanSPUC cohort in 2029-2024. After excluding participants with incomplete risk assessment questionnaire (n=9,587) and those with risk assessment results showing not high risk for lung cancer (n=123,591) and smoking female (n=2,071), 88,797 remaining participants of high risk for lung cancer were included in the final analyses. The characteristics of the high-risk population of lung cancer are presented in **Table 1**, including demographic characteristics, lifestyle factors, baseline comorbidity, and female reproductive factors. Overall, the enrolled population comprised a nearly equal proportion of males and non-smoking females (50.9% vs. 49.1%). Among males, 6,988 cases (15.5%) were non-smokers. The mean age was 61.6 years (SD = 6.7 years), and the majority (86.3%) were between 51 and 70 years old.

**Table 1:** Baseline characteristic of the study population.

Characteristic	Participants at high risk for lung cancer, No. (%)		*P* value
	Nonsmoking female (n=43,604)	Male (n=45,193)	
Demographic characteristics			
Age,y			<0.001
45-50	1599 (3.7)	1051 (2.3)	
51-55	9709 (22.3)	7230 (16.0)	
56-60	10891 (25.0)	9295 (20.6)	
61-65	9609 (22.0)	11353 (25.1)	
66-70	8020 (18.4)	10569 (23.4)	
71-74	3776 (8.7)	5695 (12.6)	
BMI			<0.001
<18.5	1468 (3.4)	1296 (2.9)	
18.5-24.0	25232 (57.9)	23244 (51.4)	
24.0-28.0	13640 (31.3)	17088 (37.8)	
>=28.0	3264 (7.5)	3565 (7.9)	
Educational level			<0.001
Primary school or less	24791 (56.9)	25030 (55.4)	
Junior or senior high school	17134 (39.3)	18069 (40.0)	
Undergraduate degree or more	1679 (3.9)	2094 (4.6)	
Lifestyle factors			
Occupational exposure to hazardous substances			<0.001
No	30174 (72.2)	28056 (64.2)	
Yes	11606 (27.8)	15649 (35.8)	
Smoking status			
Never	43,604	6988 (15.5)	
Current/Former	/	38205 (84.5)	
Passive smoking			/
No	12296 (28.2)	/	
Yes	31308 (71.8)	/	
Family history of lung cancer			<0.001
No	36954 (84.8)	40735 (90.1)	
Yes	6648 (15.2)	4451 (9.9)	
Baseline comorbidity			
History of COPD			<0.001
No	35866 (82.3)	37689 (83.4)	
Yes	7738 (17.7)	7504 (16.6)	
Hypertension			0.053
No	28179 (64.6)	29487 (65.2)	
Yes	15425 (35.4)	15706 (34.8)	
Hyperlipidemia			<0.001
No	35303 (81.0)	39069 (86.4)	
Yes	8301 (19.0)	6124 (13.6)	
Diabetes			0.009
No	41121 (94.3)	42800 (94.7)	
Yes	2483 (5.7)	2393 (5.3)	
Female reproductive factors			
Age at menarche, y			/
13-16	31432 (72.9)	/	
<13 and >16	11708 (27.1)	/	
Age at menopause, y			/
43-52	28909 (76.2)	/	
<43 and >52	9022 (23.8)	/	
Reproductive span, y			/
30-35	18884 (50.0)	/	
<30 and >35	19199 (50.0)	/	
Age at first live birth, y			/
20-25	26943 (73.4)	/	
<20 and >25	9768 (26.6)	/	
Duration of breastfeeding, months			/
7-12	22539 (59.9)	/	
<7 and >12	15081 (40.1)	/	

BMI, body mass index (calculated as weight in kilograms divided by height in meters squared); COPD, chronic obstructive pulmonary disease, includes chronic bronchitis and emphysema. Overall, there were 3312 participants without information on Occupational exposure to hazardous substances, 9 participants without information on FHLC, 464 participants without information on Age at menarche, 5673 participants without information on Age at menopause, 5521participants without information on Reproductive span, 6893 participants without information on Age at first live birth, 5984 participants without information on Duration of breastfeeding.

### 3.2 Comparative benefits of lung cancer screening in non-smoking female, non-smoking male, and smoking male

**Figure 2:**
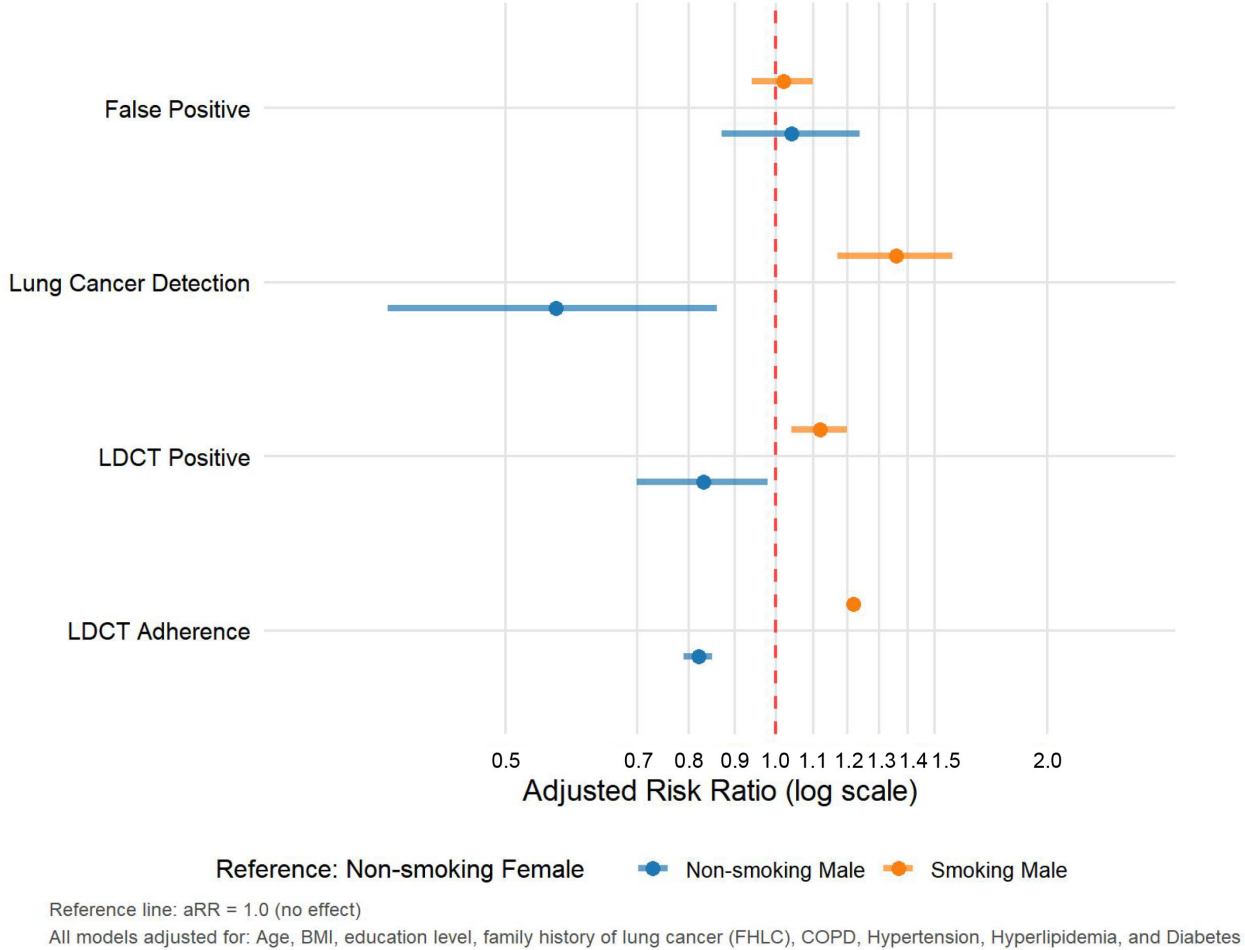
Comparison of LDCT lung cancer screening effectiveness between non-smoking/smoking males and non-smoking females.

Among 88,797 individuals identified as high-risk for lung cancer, 54,333 completed LDCT screening as recommended by the program, yielding an overall participation rate of 61.2%. Compared to non-smoking females, both non-smoking males and smoking males demonstrated distinct screening patterns. Non-smoking males showed significantly lower LDCT adherence (aRR=0.82, 95% CI 0.79-0.85), reduced positive rates (aRR=0.83, 95% CI 0.70-0.98), and substantially decreased lung cancer detection (aRR=0.57, 95% CI 0.37-0.86), while maintaining comparable false-positive rates (aRR=1.04, 95% CI 0.87-1.24) (**Figure 2**). In contrast, smoking males exhibited significantly higher adherence (aRR=1.22, 95% CI 1.20-1.24), elevated positive rates (aRR=1.12, 95% CI 1.04-1.20), and increased lung cancer detection (aRR=1.36, 95% CI 1.17-1.57), with similar false-positive rates (aRR=1.02, 95% CI 0.94-1.10) relative to non-smoking females (**Figure 2**). Collectively, compared to non-smoking females, screening outcomes varied substantially by male smoking status: non-smoking males demonstrated consistently lower rates across all metrics, while smoking males showed significantly elevated adherence, positive rates, and lung cancer detection.

### 3.3 Risk stratification for lung cancer in non-smoking female

In this study, the definition of hormonal abnormalities as having ≥2 reproductive factor abnormalities was established as the optimal cutoff value following sensitivity analyses. Through detailed sensitivity analyses comparing three definitions of hormonal abnormalities (≥1, ≥2, and ≥3 abnormalities), the rationality of the ≥2 definition was validated. Results demonstrated that the original definition (≥2 abnormalities) performed optimally across multiple metrics: superior risk discrimination (RR = 1.41 vs. 1.12/1.34 for ≥1/≥3 definitions, respectively), balanced population coverage (55.0% vs. 85.9%/23.7%), and improved model goodness-of-fit. These findings collectively confirm the robustness and epidemiological appropriateness of using ≥2 abnormalities as the threshold for risk stratification.

**Figure 3:**
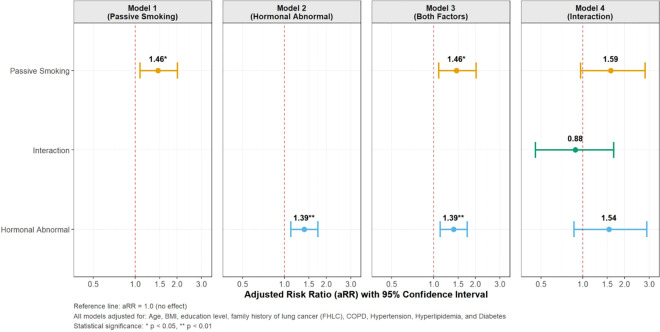
Assessment of independent risk factors for risk enrichment of lung cancer among non-smoking females.

**Figure 3** showed the results of multivariable robust (modified) Poisson regression analyses identifying risk factors for lung cancer detection among non-smoking females. Four models were constructed: Model 1 and Model 2 separately adjusted for passive smoking and hormonal abnormalities, respectively. Passive smoking was associated with a 46% significantly increased risk of lung cancer, while hormonal abnormalities were associated with a 39% elevated risk. The fully adjusted Model 3 confirmed that both passive smoking and hormonal abnormalities are independent risk factors for lung cancer in non-smoking females. Model 4 evaluated the interaction between passive smoking and hormonal abnormalities, revealing no significant multiplicative interaction (aRR=0.89, 95% CI 0.46-1.66, p=0.710). Based on these two factors, non-smoking females were stratified into distinct four risk groups: low risk group (with neither factors), medium risk group (with passive smoking only), medium risk group (with hormonal abnormalities only) and high risk group (with both factors).

### 3.4 Comparative benefits of lung cancer screening in four non-smoking female groups and male groups (all male group and smoking male group)

**Figure 4:**
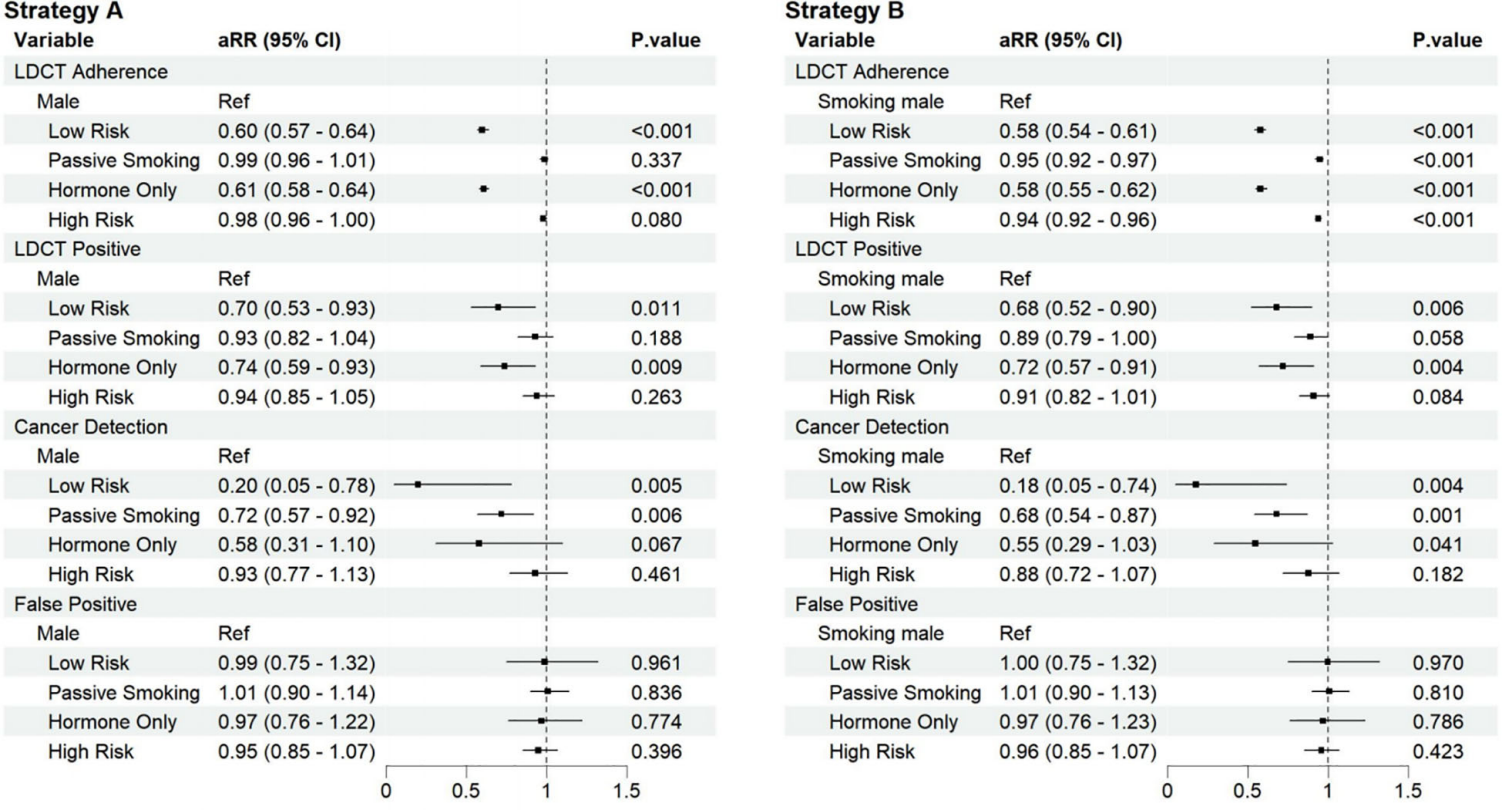
Comparison of key screening metrics between risk-stratified non-smoking female subgroups and male (Strategy A: all male; Strategy B: smoking male).

**Figure 4** compared four key screening indicators between distinct risk subgroups of non-smoking females and male groups. After adjusting for age, BMI, educational level, passive smoking, occupational exposure to hazardous substances, history of tuberculosis; history of COPD; family history of lung cancer, hypertension; hyperlipidemia and diabetes. Compared to all males, most non-smoking female subgroups showed significantly lower screening metrics. However, the high-risk subgroup with both passive smoking and hormonal abnormalities demonstrated no statistically significant difference in lung cancer detection (aRR=0.93, 95% CI 0.77-1.13) and false-positive rates (aRR=0.95, 95% CI 0.85-1.07). Similarly, the hormonal abnormalities-only subgroup showed non-significant differences in lung cancer detection (aRR=0.58, 95% CI 0.31-1.10).

When compared specifically to smoking males, similar patterns emerged. The high-risk subgroup with both factors maintained no statistically significant difference in lung cancer detection (aRR=0.88, 95% CI 0.72-1.07) and false-positive rates (aRR=0.96, 95% CI 0.85-1.07), while the hormonal abnormalities-only subgroup also showed non-significant differences in detection rates (aRR=0.55, 95% CI 0.29-1.03). Collectively, these findings identify a well-defined high-risk subgroup of non-smoking females characterized by combined passive smoking and hormonal abnormalities. This subgroup exhibits screening outcomes for both lung cancer detection and false-positive rates that show no statistically significant difference compared to those in male populations.

## 4 Discussion

This study compared the effectiveness of a one-off LDCT screening between non-smoking females and males at high risk for lung cancer. Key metrics included LDCT adherence, positive rate, lung cancer detection rate, and false-positive rate. Non-smoking females demonstrated significantly lower rates than smoking males across all four indicators, suggesting a limitation of current screening criteria when applied to females. Prompted by this finding, we further employed a risk-factor enrichment strategy, which revealed significant heterogeneity within the non-smoking female population. Multivariable modified Poisson regression identified passive smoking and hormonal abnormalities as independent risk factors for lung cancer in non-smoking females. Risk stratification based on these two factors revealed that non-smoking females exhibiting both risk factors showed no significant differences in LDCT adherence, positive rate, lung cancer detection rate, or false-positive rate compared to males, including smoking males. These findings suggest that non-smoking females with both passive smoking exposure and hormonal abnormalities may benefit from LDCT screening in a manner comparable to male counterparts.

Our study revealed substantial disparities in LDCT screening adherence among high-risk individuals. Non-smoking females demonstrated significantly lower adherence rates (56.6%) compared to smoking males (69.4%), with non-smoking males showing the lowest participation (45.4%). The adjusted analysis confirmed these patterns, indicating significantly reduced adherence among non-smoking males (aRR=0.82, 95% CI 0.79-0.85) and elevated adherence among smoking males (aRR=1.22, 95% CI 1.20-1.24) relative to non-smoking females. These disparities may be partly attributable to prevailing health beliefs, as lung cancer has long been predominantly associated with smoking and male gender (18). Such perceptions may lead non-smoking females and particularly non-smoking males to underestimate their personal risk, resulting in lower participation rates. In addition to risk perception, differences in health-seeking behavior between genders and systemic barriers such as logistical challenges or screening promotions that are less relevant to non-smokers may also contribute to these disparities. Notably, the overall LDCT adherence across this high-risk cohort demonstrates substantial improvement over rates reported in earlier national screening programs. The observed rates exceed those from the 2013–2018 CanSPUC in Henan Province (40.2%) (19), Zhejiang Province (48.2%) (20), and the national average during that period (35.6%) (21). This marked increase likely reflects growing public awareness of cancer screening and optimized organization and implementation of the current screening program.

Male lung cancer is primarily driven by tobacco exposure (22), often manifesting as central squamous cell carcinoma with more apparent imaging features (23–25). In contrast, lung cancer in non-smoking females may be driven by genetic predisposition, hormonal influences, or other environmental factors, and often exhibits an indolent biological behavior characterized by subtle early-stage radiological findings such as ground-glass nodules (5). This pathological profile likely contributed to their lower positive rate and lung cancer detection rate. The high false-positive rate observed in our study is consistent with the established pattern in initial LDCT screening of high-risk populations, as evidenced by a false-positive rate of 96.2% in the first round of the NLST (6). The concurrently lower false-positive rate observed in non-smoking females indicates higher screening specificity, suggesting that current criteria capture numerous non-high-risk individuals. These findings underscore the necessity of risk stratification strategies to refine screening target populations.

In this study, multivariable modified Poisson regression analysis demonstrated that both passive smoking and hormonal abnormalities independently increased lung cancer risk among high-risk non-smoking females, with no evidence of interaction between these factors. The IARC and the U.S. National Institutes of Health have classified secondhand smoke (SHS) as a Group 1 carcinogen (4). A meta-analysis by Ni et al. reported a pooled relative risk of 1.40 (95% CI: 1.08-1.82) for lung cancer attributable to passive smoking, and demonstrated a significant dose-response relationship, with risk escalating in tandem with increasing duration, intensity, and cumulative years of passive smoking (26). However, the underlying biological mechanisms through which secondhand smoke contributes to lung carcinogenesis require further investigation. Compared with lung cancer in males with a smoking history, lung cancer in non-smoking females exhibits distinct epidemiological and biological characteristics. Recent reviews highlight that estrogen, particularly through estrogen receptor beta (ERβ), plays a pivotal role in shaping a pro-tumor microenvironment in non-small cell lung cancer (NSCLC), influencing the function of various immune cells and contributing to tumor progression and immunotherapy response disparities, which provides a plausible biological basis for the hormonal associations observed in epidemiological studies (31). Previous evidence from the UK Biobank prospective cohort identified that early menopause, a shorter reproductive lifespan, and a younger age at first birth were associated with an elevated risk of lung cancer in females (27). Subsequently, a large prospective investigation from the China Kadoorie Biobank, which focused specifically on female non-smokers, revealed a distinct pattern of risk associated with exogenous hormone exposure and breastfeeding. This later study identified that oral contraceptive use was associated with an increased risk of lung cancer, whereas a longer duration of breastfeeding per child was associated with a decreased risk (16). Nevertheless, these findings are derived from observational studies with heterogeneous populations and limited hormonal measurements. Future research should prioritize prospective designs with standardized endocrine assessments to address these limitations.

The study demonstrated that non-smoking females with both passive smoking and hormonal abnormalities showed no statistically significant differences in all four key LDCT screening metrics compared to males, including smoking males. This indicates that this specific female subgroup not only carries a lung cancer risk comparable to males but also achieves similar screening effectiveness with LDCT. These findings are supported by a meta-analysis of 13 Asian studies, which revealed equivalent diagnostic yield between non-smoking females and high-risk former-smoking males (RR = 1.02, 95% CI: 0.94-1.11), along with significantly higher early-stage detection (82.4% VS. 50.6%) and lower mortality (HR = 0.59, 95% CI: 0.42-0.83) among non-smokers (28). Therefor, we recommend that this well-defined female subgroup should be considered for LDCT screening recommendations similar to those for male populations. Additionally, this well-defined subgroup offers concrete variables that could be integrated into AI-driven screening tools, to automate the efficient identification of high-risk individuals in clinical and public health (29, 30).

Limitations of this study should be noted. First, as all participants were recruited exclusively from Zhejiang Province, the generalizability of our findings to other regions, ethnic groups, or socioeconomic contexts may be limited. Second, clinical follow-up for diagnosed lung cancer cases remains ongoing; therefore, comprehensive disease characteristics such as complete cancer staging data are not yet available for analysis. Third, the classification of hormonal abnormalities was based largely on self-reported reproductive history, which is subject to potential recall bias. Fourth, we lacked data on genetic predisposition and specific tobacco product types, which represent important unmeasured confounders. Although multivariable adjustment strengthens the internal validity of our estimates, we acknowledge the potential for residual confounding due to other unmeasured or imperfectly measured factors, such as indoor air pollution. Future studies should aim to integrate direct biochemical assessments of hormone levels, genetic profiling, and detailed smoking histories to validate and improve the accuracy of this risk factor categorization.

In conclusion, current lung cancer screening criteria show limited applicability to female populations, as demonstrated by the significantly lower LDCT adherence, positive rates, and detection rates observed in non-smoking females compared with smoking males. Crucially, we identified a well-defined subgroup of non-smoking females characterized by the combined presence of passive smoking exposure and hormonal abnormalities. This subgroup exhibited screening outcomes that showed no statistically significant difference from those observed in males. This finding provides empirical evidence supporting the development of more precise, risk-stratified screening criteria that better account for sex-specific differences in lung cancer risk and clinical presentation.

## Data Availability

The raw data supporting the conclusions of this article will be made available by the authors, without undue reservation.
